# The factors associated with the quality of life among postmenopausal women

**DOI:** 10.1186/s12905-021-01361-x

**Published:** 2021-05-18

**Authors:** Majid Barati, Hakimeh Akbari-heidari, Elham Samadi-yaghin, Ensiyeh Jenabi, Hanieh Jormand, Naser Kamyari

**Affiliations:** 1 Autism Spectrum Disorders Research Center, Hamadan University of Medical Sciences, Hamadan, Islamic Republic of Iran; 2 Students Research Committee, Hamadan University of Medical Sciences, Hamadan, Islamic Republic of Iran

**Keywords:** Quality of life, Health, Women, Postmenopause, Smoking, Dietary supplements

## Abstract

**Objective:**

The present work aimed at determining the prevalence of menopausal symptoms and factors associated with the Quality of life among postmenopausal women.

**Materials and methods:**

This cross-sectional work was carried out on 270 postmenopausal females referring to health centers of Hamadan city chosen by stratified random sampling. A questionnaire of the Menopausal Quality of Life Questionnaire (MENQOL) was used as the data collection method. Although, the Mann–Whitney test and the Kruskal–Wallis tests were used to compare MENQOL item scores. The significance level of statistical tests was regarded as less than 0.05.

**Results:**

The mean MENQOL Score in menopausal was 2.45 ± 1.04. Also, vasomotor symptoms had the highest score, and sexual symptoms had the lowest score rather than other dimensions. There was a significant association between the total menopausal quality of life score and job, economy status, smoking, exercise, supplemental Omega-3 s intake, and Postmenopausal stage (*p* < 0.01). As smokers, women had increasing levels of bother experienced from the MENQOL symptom than non-smokers (mean = 3.67 ± 0.85 vs. 2.36 ± 0.99; *p* < 0.001). While the MENQOL scores for menopausal females who exercised more often (mean = 1.56 ± 0.7) had lower than those who exercised less than 3 times per week (mean = 3.27 ± 0.9; *p* < 0.001). However the lowest score was menopausal females who had taking supplemental Omega-3 s than those who hadn't taken it (mean = 2.15 ± 1.06 vs. 2.65 ± 0.97; *p* < 0.001). Though women who had postmenopausal stage less than 5 years stage (mean = 2.28 ± 0.87) had significantly lower MENQOL scores from those who had postmenopausal stage 5 or more years (mean = 2.63 ± 1.16; *p* < 0.001).

**Conclusion:**

Based on the results, vasomotor symptoms were the most dominant symptom. Therefore, it is necessary to improve physical activity levels, focusing on job status, recommend taking an omega 3 s supplement, and planning education and promotion intervention for cessation or prevention of smoking among postmenopausal women to increase the MENQOL is essential.

## Introduction

Due to a decrease in the estrogen level of blood, menopause causes several symptoms and complications in women, including termination of reproductive capacity resulting in the complete cessation of ovarian function [1, 2], vasomotor instability, decreased psychological function, forgetfulness, and vaginal and urinary tract infections [3]. Regarding cultural and ethnic differences, the severity and frequency of these symptoms vary in different countries [1, 4]. Factors such as genetics, dietary habits, level of activity, and daily exercises cause differences in the natural age of menopausal in various communities. Low self-efficacy, extensive cultural conflicts, and socioeconomic inequalities, belief and gender inequalities, knowledge of the menopausal process, and stressors are the most significant factors influencing the menopausal Quality of life (QOL) [5]. As a result, vasomotor symptoms show a higher prevalence in African American and Western women than Asian women [6].

The World Health Organization (WHO) has defined the QOL as people's insights into their life status within the value and cultural systems where they live and concerning their expectations, goals, concerns, and standards [7]. The multidimensional QOL has been accepted nowadays [6]. Changes in life expectancy have led to an increase in post-menopausal life expectancy in women. Each woman spends at least one-third of her life during the menopausal period [8]. Statistics show an increasing number of postmenopausal women worldwide. In 1998, more than 477 million women were living in the postmenopausal period, and this rate will reach 1.1 billion people by 2025 [9]. According to some newer statistics in the US, 6000 people reach menopausal age every day, and about 50 million will be menopausal over the next decade. Iran is also expected to have about 5 million women with menopausal by 2021, leading to demographic changes and population aging [10].

The mean age of menopausal in Iran is lower than that in other countries due to several factors. Based on the results of various studies in Iran, the QOL of menopausal females has been reported at a moderate level [11, 12]. Women make up half of Iran's population. Therefore, gaining information and knowledge of their conditions in various dimensions and planning to reform their conditions to affect society and family health seems necessary [13]. Also, given the role of women as the key members of family health and transmitting the model of education and promoting a healthy lifestyle to the next generation, acquiring knowledge of this period's symptoms and enhancing their QOL is of great necessity [5, 14]. Therefore, the current work was conducted to assess the Factors related to MENQOL among postmenopausal women in the health center of Hamadan City, Iran.

## Materials and methods

### Study type

The current descriptive-analytical cross-sectional work was performed on 270 women aged 45–60 years covered by Hamadan health centers in 2018.

### Participants

Using the data of previous studies [15] and the formula for determining the sample size [16], the minimum sample size was determined at 250 people. Considering 5% error and 80% power and considering the effect of cluster sampling design, the sample size was determined to be 270 postmenopausal women. In the present research, the participants were selected using the stratified sampling method. Accordingly, after listing health centers in Hamadan, some of these centers were selected using a cluster sampling method. Then, after gaining permits from the centers' authorities, the samples were selected from the population covered by each center. Finally, some women aged 45–60 years covered by the centers were selected, and the data were collected. The inclusion criteria were being 45–60 years, having normal menopausal, living in Hamadan, lack of alternative hormone therapy in the past six months, no history of hysterectomy, and lack of known systemic illness. The exclusion criteria also included non-responding to all items of the questionnaire and having any physical and mental illness based on their self-report.

### Statement

All methods were performed in accordance with relevant guidelines and regulations. The word informed consent was obtained from all women; they were informed about the confidentiality of the information and the project's purpose, and only if they would like, they were enrolled in the study. The Ethics Committee approved this study with all consent process at Hamadan University of Medical Sciences.

### Survey questionnaire

The data collection tool used in this study consists of two parts completed by the women through the self-reporting method. The first part includes demographic information such as age, education level, economic status, marital status, smoking, job, physical activity, and supplemental Omega-3 s intake. Also, the second section includes the Menopausal Quality of Life Questionnaire (MENQOL). MENQOL designed by Hilditch et al. [17, 18] that assesses the QOL in 4 dimensions: (1) physical dimension with 16 questions (questions 11–26); (2) the psychosocial dimension with 7 questions (questions 4–10); (3) the sexual dimension with 4 questions (questions 27–29); and (4) vasomotor dimension with 3 questions (questions 1–3), respectively. This scale, which also was used in this study, is adopted to the Persian language and is appropriate for Iranian culture [17, 19]. The validity and reliability of the questionnaire have been confirmed in several similar studies. For example, the reliability of the MENQOL questionnaire was assessed using the test–retest based on intraclass correlation coefficients (ICCs). The coefficients were 0.79, 0.68, 0.92, and 0.77 for the psychological, physical, sexual, and vasomotor domains, respectively [6]. This questionnaire was on a seven-point Likert scale ranging from 0 to 7. A “zero” is identical that the woman has not experienced this specific symptom in the previous month; Score “one” demonstrates that the woman experienced the symptom, but it did not bother her; Scores “two” through “seven” demonstrate an increasing level of the bothering symptom, so that these scores indicate increasing levels of bother experienced from the symptom and correspond to the ‘‘1’’ through ‘‘6’’ checkboxes on the MENQOL [6, 20]. It is essential to be mention, with increasing MENQOL scores, levels of bother experienced from the symptom are increased as well.

According to the definition of WHO, menopausal health was considered menstruation cessation without medication for at least 12 months [4]. Before the study, necessary explanations were provided for the subjects regarding the study's objectives, and their informed consent was obtained. The subjects were selected and included in the study in terms of exclusion and inclusion criteria. The demographic characteristics questionnaire and QOL questionnaire of postmenopausal females were completed through a face-to-face interview.

The duration of the interview was around 25–30 min.

### Analysis of statistical data

SPSS V.23 was used to analyze the collected data. The Mann–Whiney test and the Kruskal–Wallis test were used to compare different dimensions of QOL according to individual characteristics. The significance level of statistical tests was regarded as less than 0.05. The Kolmogorov–Smirnov test showed that data were not normally distributed, the Mann–Whiney U test and the Kruskal–Wallis test were used to compare MENQOL item scores.

## Results

### Demographic characteristics

The mean age of the women was 52.19 ± 3.98 years with a range of 45–60 years old. About 25.2% had an elementary education and 5.6% have university education; 77.8% were married and 14.8 were widow; about 11% had Clerk and 88.9% were housewives; 7.4% were smoking, and 59.6% had medium economic status; 47.8% usually exercise 3–5 times a week; 38.9% were taking supplemental Omega-3 s; 50.4% had been postmenopausal for less than 5 years and 40.6% for more than 5 years. Other demographic and characteristics of participants are presented in Table 1.

**Table 1:** Characteristics of participants (N = 270)

Variables	Frequency n	(%)
*Age*		
< = 55	180	66.7
> 55	90	33.3
*Education*		
Illiterate	68	25.2
Elementary	104	38.5
Secondary	55	20.4
Diploma	25	10.4
University	15	5.6
*Marital status*		
Married	210	77.8
Single	11	4.1
Divorce	9	3.3
Widow	40	14.8
*Job*		
Housewife	240	88.9
Clerk	30	11.1
*Economy status*		
Low	79	29.3
Mid	161	59.6
High	30	11.1
*Smoking*		
Yes	20	7.4
No	250	92.6
*Exercise (times/week)*		
< 3	72	29.7
3–5	129	47.5
> 5	69	25.6
*Supplemental Omega-3 s intake*		
Yes	105	38.9
No	165	61.1
*Postmenopausal stage*		
Less than 5 years ago	136	50.4
5 or more years	134	40.6

### The menopausal quality of life, and associated factors

According to the association between Quality of Life and demographic variables, there was a significant association between the total QoL score and job, economy status, smoking, exercise, supplemental Omega-3 s intake, and postmenopausal stage (*p* < 0.01). As smokers, women had increasing levels of bother experienced from the symptom than non-smokers (mean = 3.67 ± 0.85 vs. 2.36 ± 0.99; *p* < 0.001). While the MENQOL scores for menopausal females who exercised more often (mean = 1.56 ± 0.7) had lower than those who exercised less than 3 times per week (mean = 3.27 ± 0.9; *p* < 0.001). However the lowest score was menopausal females who had taking supplemental Omega-3 s than those who hadn't taken it (mean = 2.15 ± 1.06 vs. 2.65 ± 0.97; *p* < 0.001). Though women who had postmenopausal stage less than 5 years stage (mean = 2.28 ± 0.87) had significantly lower MENQOL scores from those who had postmenopausal stage 5 or more years (mean = 2.63 ± 1.16; *p* < 0.001).

Besides, there was a significant association between the vasomotor dimension of MENQOL and economy status, smoking, exercise, supplemental Omega-3 s intake (*p* < 0.01).

Moreover, high-socioeconomic status (SES) women had significantly lower vasomotor scores than those who had postmenopausal low-socioeconomic status (SES) (mean = 2.29 ± 1.81 vs. 3.34 ± 1.97; *p* < 0.001). Smokers (mean = 4.42 ± 1.94) increasing levels of bother experienced from the vasomotor symptom than non-smokers (mean = 2.68 ± 1.89; *p* < 0.001). While menopausal females who exercised more often had vasomotor scores (mean = 1.94 ± 1.66) lower than those who exercised less than 3 times per week (mean = 3.63 ± 1.91; *p* < 0.001). However menopausal females who had taking supplemental Omega-3 s more often had mean vasomotor scores lower than those who hadn't taken it (mean = 2.38 ± 1.87 vs. 3.08 ± 1.94; *p* < 0.001).

Although, there was a significant association between the psychosocial dimension of MENQOL and education, job, economic status, smoking, exercise, supplemental Omega-3 s intake, postmenopausal stage (*p* < 0.01), and marital status (*p* < 0.05). Through, educated women (mean = 1.35 ± 1.07) had significantly lower psychosocial scores than illiterate women (mean = 2.53 ± 1.18; *p* < 0.001). Clerk women (mean = 1.67 ± 1.24) had significantly lower psychosocial scores from housewife women (mean = 2.68 ± 1.07; *p* < 0.05). High-socioeconomic status (SES) women had significantly lower psychosocial scores than those who had postmenopausal low-socioeconomic status (SES) (mean = 1.32 ± 0.93 vs. 2.94 ± 1.25 *p* < 0.001). Smokers (mean = 4.31 ± 0.68) increasing levels of bother experienced from the psychosocial symptom than non-smokers (mean = 2.1 ± 1.17; *p* < 0.001). While menopausal females who exercised more often had psychosocial scores (mean = 3.68 ± 0.95) lower than those who exercised less than 3 times per week (mean = 0.85 ± 0.55); *p* < 0.001). However menopausal females who had taking supplemental Omega-3 s more often had mean psychosocial scores lower than those who hadn't taken it (mean = 1.99 ± 1.29 vs. 2.43 ± 1.24; *p* < 0.001). Also, married women (mean = 2.14 ± 1.26) had significantly lower psychosocial scores than divorce women (mean = 2.94 ± 1.15; *p* < 0.05).

While there was a significant association between the physical dimension of MENQOL and economy status, smoking, exercise, supplemental Omega-3 s intake, postmenopausal stage (*p* < 0.01), and job (*p* < 0.05). By way of, Clerk women (mean = 2.22 ± 0.87) had significantly lower physical scores than housewife women (mean = 2.68 ± 1.07; *p* < 0.05). High-socioeconomic status (SES) women had significantly lower physical scores than those who had postmenopausal low-socioeconomic status (SES) (mean = 1.88 ± 0.83 vs. 3.02 ± 1.08 *p* < 0.001). Smokers (mean = 3.46 ± 0.66) increasing levels of bother experienced from the physical symptom than non-smokers (mean = 2.57 ± 1.06; *p* < 0.001). While menopausal females who exercised more often had physical scores (mean = 1.79 ± 0.9) lower than those who exercised less 3 times per week (mean = 2.37 ± 0.92); *p* < 0.001). However menopausal females who had taking supplemental Omega-3 s more often had mean physical scores lower than those who hadn't taken it (mean = 2.38 ± 1.87 vs. 2.79 ± 0.98; *p* < 0.001).

However, there was a significant association between the sexual dimension of MENQOL and, job, marital status (*p* < 0.01), education, and exercise (*p* < 0.05). By way of, Clerk women (mean = 1.17 ± 1.49) had significantly lower sexual scores than housewife women (mean = 2.23 ± 1.79; *p* < 0.001). Also, married woman (mean = 2.67 ± 1.60) had significantly higher sexual scores from widow women (mean = 0.28 ± 0.94; *p* < 0.001). Educated women (mean = 1.40 ± 1.69) had significantly lower sexual scores than women with low levels of education (mean = 2.44 ± 1.50; *p* < 0.001). While menopausal females who exercised more often had sexual scores (mean = 1.65 ± 1.54) lower than those who exercised less 3 times per week (mean = 2.43 ± 2.05); *p* < 0.05) (Table 2).

**Table 2:** Association between dimension quality of life and demographic variables in postmenopausal women

Variables	Vasomotor dimension	Psychosocial dimension	Physical dimension	Sexual dimension	Total quality of life
	Mean ± SD	Mean ± SD	Mean ± SD	Mean ± SD	Mean ± SD
*Age*					
< = 55	2.95 ± 1.86	2.23 ± 1.26	2.56 ± 1.06	2.18 ± 1.78	2.48 ± 1.05
> 55	2.53 ± 2.09	2.32 ± 1.31	2.77 ± 1.05	1.99 ± 0.19	2.40 ± 1.01
*p*-value	0.094	0.614	0.132	0.425	0.555
*Education*					
Illiterate	3.06 ± 2.07	2.53 ± 1.18	2.87 ± 1.11	1.58 ± 1.86	2.51 ± 1.1
Elementary	2.91 ± 2.04	2.41 ± 1.42	2.64 ± 1.07	2.41 ± 1.83	2.59 ± 1.09
Secondary	2.40 ± 1.67	2.00 ± 1.15	2.45 ± 1.02	2.44 ± 1.50	2.32 ± 0.86
Diploma	2.82 ± 1.65	2.05 ± 0.99	2.58 ± 0.94	2.06 ± 1.78	2.38 ± 0.89
University	2.44 ± 2.1	1.35 ± 1.07	2.27 ± 1.0	1.40 ± 1.69	1.87 ± 1.05
*p* value^a^	0.354	0.004**	0.131	0.01*	0.09
*Marital status*					
Married	2.73 ± 1.88	2.14 ± 1.26	2.56 ± 1.06	2.67 ± 1.60	2.53 ± 1.05
Single	2.97 ± 1.93	2.82 ± 1.28	2.94 ± 1.06	0	2.18 ± 0.76
Divorce	3.67 ± 1.37	2.94 ± 1.15	2.97 ± 1.0	0	2.39 ± 0.51
Widow	2.97 ± 2.34	2.59 ± 1.28	2.86 ± 1.04	0.28 ± 0.94	2.18 ± 1.06
*p* value^a^	0.496	0.027*	0.196	0.001**	0.19
*Job*					
Housewife	2.86 ± 1.96	2.33 ± 1.27	2.68 ± 1.07	2.23 ± 1.79	2.53 ± 1.03
Clerk	2.40 ± 1.78	1.67 ± 1.24	2.22 ± 0.87	1.17 ± 1.49	1.87 ± 0.89
*p* value^b^	0.221	0.007**	0.023*	0.002**	0.001**
*Economy status*					
Low	3.34 ± 1.97	2.94 ± 1.25	3.02 ± 1.08	1.71 ± 1.93	2.76 ± 1.04
Mid	2.10 ± 1.91	2.73 ± 1.19	2.58 ± 1.0	2.31 ± 1.72	2.41 ± 1.0
High	2.29 ± 1.81	1.32 ± 0.93	1.88 ± 0.83	2.14 ± 1.66	1.91 ± 0.92
*p* value^a^	0.009**	0.001**	0.001**	0.056	0.001**
*Smoking*					
Yes	4.42 ± 1.94	4.31 ± 0.68	3.46 ± 0.66	2.47 ± 2.17	3.67 ± 0.85
No	2.68 ± 1.89	2.1 ± 1.17	2.57 ± 1.06	2.09 ± 1.76	2.36 ± 0.99
*p* value^b^	0.001**	0.001**	0.001**	0.364	0.001**
*Exercise (times/week)*					
< 3	3.63 ± 1.91	3.68 ± 0.95	2.37 ± 0.92	2.43 ± 2.05	3.27 ± 0.9
3–5	2.82 ± 1.91	2.22 ± 0.75	2.68 ± 0.88	2.19 ± 1.73	2.48 ± 0.86
> 5	1.94 ± 1.66	0.85 ± 0.55	1.79 ± 0.9	1.65 ± 1.54	1.56 ± 0.7
*p* value^a^	0.001**	0.001**	0.001**	0.029*	0.001**
*Supplemental Omega-3 s intake*					
Yes	2.38 ± 1.87	1.99 ± 1.29	2.38 ± 1.12	1.85 ± 1.67	2.15 ± 1.06
No	3.08 ± 1.94	2.43 ± 1.24	2.79 ± 0.98	2.28 ± 1.85	2.65 ± 0.97
*p* value^b^	0.004**	0.005**	0.002**	0.055	0.001**
*Postmenopausal stage*					
Less than 5 years ago	2.77 ± 1.74	1.82 ± 0.96	2.45 ± 1.00	2.08 ± 1.60	2.28 ± 0.87
5 or more years	2.85 ± 2.14	2.70 ± 1.41	2.82 ± 1.08	2.15 ± 1.98	2.63 ± 1.16
*p* value^b^	0.764	0.001**	0.001**	0.746	0.006**

^a^*p* values based on the Kruskal–Wallis H-test

^b^*p* values based on the Mann–Whitney U-test

**The level of significance was set at *p* < .01, *The level of significance was set at *p* < .05

### Frequency distribution and scores of menopausal items

Generally, the most dominant symptom, reported by 91.1% of the sample, was aching in muscles and joints ache. Besides, vasomotor symptoms with 2.80 ± 1.94 had the highest score, and sexual symptoms, with 2.12 ± 1.79 had the lowest score among the other dimensions. The mean Score of MENQOL in menopausal women was 2.45 ± 1.04. In the other words, we found that vasomotor symptoms had the highest mean scores and sexual symptoms had the lowest scores in all domains (Table 3).

**Table 3:** Frequency distribution and scores of menopausal items

Symptom	No. (%)	Mean ± SD
Vasomotor dimension Hot flushes or flashes	205 (75.9)	3.46 ± 2.40
Night sweats	160 (59.3)	2.61 ± 2.48
Sweating	160 (59.3)	2.36 ± 2.32
Total vasomotor dimension		2.80 ± 1.94
Psychosocial dimension Dissatisfied with personal life	116 (43.0)	1.55 ± 1.98
Feeling anxious or nervous	226 (83.7)	3.49 ± 1.99
Poor memory	185 (68.5)	2.50 ± 2.9
Accomplishing poor memory	164 (60.7)	2.25 ± 2.01
Feeling depressed, down, or blue	157 (58.1)	2.23 ± 2.23
Being impatient with other people	144 (53.3)	2.17 ± 2.26
The feeling of wanting to be alone	106 (39.3)	1.64 ± 2.22
Total psychosocial dimension		2.26 ± 1.27
Physical dimension Flatulence (wind) or gas pains	148 (54.8)	2.36 ± 2.41
Aching in muscles and joints	246 (91.1)	4.38 ± 1.87
Feeling tired or worn out	231 (85.6)	3.51 ± 1.88
Difficulty sleeping	184 (68.1)	3.07 ± 2.43
Aches in back of neck or head	165 (61.1)	2.71 ± 2.44
The decrease in physical strength	233 (86.3)	3.53 ± 1.81
Decrease in stamina	206 (76.3)	3.06 ± 2.05
Lack of energy	237 (87.8)	3.53 ± 1.70
Dry skin	147 (54.4)	2.17 ± 2.24
Weight gain	108 (40)	1.47 ± 2.05
Increased facial hair	97 (35.9)	1.23 ± 1.85
Changes in appearance, texture, or tone of the skin	187 (69.3)	2.40 ± 1.87
Feeling bloated	76 (28.1)	1.04 ± 1.86
Low backache	220 (81.5)	3.81 ± 2.24
Frequent urination	133 (49.3)	2.05 ± 2.33
Involuntary urination when laughing or coughing	139 (51.5)	1.81 ± 2.02
Total physical dimension		2.63 ± 1.06
sexual dimension change in sexual desire	158 (58.5)	2.46 ± 2.31
Vaginal dryness during intercourse	79 (29.3)	1.20 ± 2.05
Avoidance of intimacy	162 (60)	2.69 ± 2.42
Total sexual dimension		2.12 ± 1.79
Total quality of life	270 (100)	2.45 ± 1.04

## Discussion

The present work was performed to determine the prevalence of menopausal symptoms among postmenopausal females and factors associated with the Quality of these women's lives in Hamadan city. Based on the obtained results, the lowest symptoms belonged to the sexual dimension, which is in line with the outcomes of the study conducted by Chedraui et al. and Parsa et al. [21, 22]. So, it can be stated that enhancing information and knowledge alongside high education level, increasing support and counseling [10], and improving adaptation and adaptive behaviors in postmenopausal periods may improve the MENQOL in the sexual dimension among this group of menopausal women.

The findings indicated a significant association between the total QoL score and job, economy status, smoking, exercise, and supplemental Omega-3 s intake. Consistent with the results of the researches reported that the MENQOL was associated with the job [6, 23, 24]. In general, employed women may have better access to health care services and adapt to postmenopausal symptoms. On the other hand, based on the results of Fallahzadeh et al., being employed, and high social interaction with other people can reduce the physical symptoms of menopausal [6]. Overall, it seems that menopausal women are more likely to have menopausal complications because of the stresses of working at home. So, it may be necessary to design and implement effective educational and promotional interventions to improve the sense of usefulness in menopausal women.

The poor economic status may cause the severity of menopausal symptoms. In this sense, Li et al. have previously reported low economic status as the main factors contributing to the incremented severity of menopausal symptoms or a longer duration of the menopausal symptoms [25]. His finding is also similar to that reported by Fallahzadeh et al. [6]. However, being employed and having high-income levels may be increased access to healthcare or the level of coping mechanisms for menopausal symptoms.

Nicotine dependence and smoking may reason the severity of menopausal Symptoms amongst postmenopausal women Smokers. These results are consistent with the funding of Copeland et al. [26]. However, cigarette smoking and hookah smoking have antiestrogenic properties, which may health risks associated with menopause symptoms.

A high physical activity level may reduce the menopausal symptom. This funding is similar to the results of other studies [27, 28]. Concerning, exercise has long been proven to be as effective as relaxation and meditation, as well as aerobic exercise, induces weight loss and increases in their hot flashes' severity and reductions in the risk of memory problems. It should be noted that exercise and physical activity in this group of women leads to increased social interactions, and group aerobic exercise leads to improved mental health and Quality of life in this group of people. So, having exercise play improvement of women's Quality of life in menopausal women.

There was an association between menopausal symptoms and supplemental Omega-3 s intake in postmenopausal women. Saimin et al. reported increasing the quantity and frequency of fish consumption will reduce the severity of menopausal symptoms [29]. Studies showed that supplemental omega-3 s could improve overall health conditions [30, 31]. So, design and planning intervention programs for a healthy lifestyle [32], for the improvement of women's Quality of life in menopausal women seems to be essential.

Additionally, in the psychosocial, physical domains, women who were in the postmenopausal stage less than 5 years ago had lower scores than those who were in the postmenopausal stage more than 5 years ago. Also similar to that reported by Fallahzadeh et al. and Karmakar et al. [6, 33]. Nevertheless, some women experience symptoms earlier during the time since menopause while some experience them at a later time. Concerning, older women might have learned to handle menopause-related symptoms over time.

In the present study, the highest MENQOL in menopausal women belonged to the vasomotor dimension, and the lowest belonged to the sexual dimension. In line with this result, several studies by Barati et al., Fallahzadeh, Chedraui, et al., Blumelet al., Whelan et al., Nisar et al. reported higher vasomotor symptoms [6, 15, 34–37]. Moreover, Reproductive hormones seem to play a significant role in this regard. In this period, dramatic changes occur in hormone levels, including reduced severe estrogen, which results in Vasomotor symptoms (VMS) such as hot flushes and night sweats. Some studies have reported an increase in the frequency of vasomotor symptoms, which is consistent with the current work results [38].

Following the outcome, physical symptoms were the most experienced and complained factors, among other dimensions in the postmenopausal period. In agreement with the findings in the current work, Ceylan et al. indicated that the most frequently experienced symptoms during this period were aching muscles and joints [39]. It seems that factors such as reproductive hormones play a major role in this regard. As a result, dramatic changes in hormones, including a severe reduction in estrogen, occur, leading to physical symptoms during this period. This result is in accord with paying attention to menopausal in health care services programs to enhance women's life expectancy seems to be useful in this field.

The major limitation of the present study is its self-reporting and descriptive cross-sectional nature. Therefore, it is recommended to investigate the subject of the present study as a longitudinal or trial study. Given the complexity of MENQOL dimensions, a qualitative study of menopausal phenomena and understanding other related factors can also be helpful.

## Conclusion

In general, the results of the present work revealed that vasomotor symptoms were the most dominant symptom. Therefore, increased life expectancy among these women will be possible by designing and implementing effective educational interventions. It is necessary to improve physical activity levels, particularly in developing countries, focusing on job status to decrease unproductive feelings, encourage social interaction skills, and enhance economic status. Simultaneously, recommend taking an omega 3 s supplement and planning education and promotion intervention for cessation or prevention smoking among postmenopausal women to increase the MENQOL is essential.

## Data Availability

The datasets used and/or analyzed during the current study available from the corresponding author on reasonable request.
